# The spatial modification effect of predictors on household level food insecurity in Ethiopia

**DOI:** 10.1038/s41598-022-23918-y

**Published:** 2022-11-11

**Authors:** Zelalem G. Dessie, Temesgen Zewotir, Delia North

**Affiliations:** 1 School of Mathematics, Statistics and Computer Science, University of KwaZulu-Natal, Durban, South Africa; 2 College of Science, Bahir Dar University, Bahir Dar, Ethiopia

**Keywords:** Health care, Nutrition, Public health, Risk factors

## Abstract

Household food insecurity remains highly prevalent in developing countries (including in Ethiopia) and it has been recognized as a serious public health problem. Several factors such as demographic, economic, social, and clinical factors influence household food insecurity, and these vary geographically. In this work, we investigate the geographical modification of the effect of several factors on chronic food insecurity. The data is from the Ethiopia socioeconomic survey conducted by the Ethiopia Central Statistics Agency (ECSA) in collaboration with the World Bank. Ethiopia socioeconomic survey is a long-term project to collect nationally representative panel survey of over 6500 households. A geo-additive model which accounts the structured and unstructured special effect was adopted to estimate household food insecurity risk factors. The study also revealed significant spatial variations on household food insecurity among administrative zones. Mainly, household living in the Sidama, Gamo Gofa, Shinille, Basketo, Wolyita, Wag Hemira, Liben, Awi, Eastern Tigray and West Harerghe zones, having higher food insecurity than the other zones in Ethiopia. Moreover, the analysis also showed that availability of credit services, proximity to service centers, average years of schooling of members of the household, and household assets are negatively associated with household food insecurity, whereas shocks, age, and dependency ratio increase the odds of a household to be food insecured. The generalized geo-additive mixed-effects model enables simultaneous modeling of spatial correlation, heterogeneity and possible nonlinear effects of covariates. Our study investigated the spatial heterogeneity of household level food insecurity, and its association with shocks, age, dependency ratio, availability of credit services, average years of schooling, and household assets. Our findings have also an important implication for planning as well as in the search for the variables that might account for the residual spatial patterns.

## Introduction

Adequate quality and quantity of food are required for the optimal health, growth and development of humans. Hence, the concern of food security for mankind has continued to be the priority agendas of leaders around the world. According to World Bank and the United Nations^1^, food security is a concept that existed when all people at all times have social, physical, and economic access to safe, sufficient, and nutritious food that meets their dietary needs for an healthy and active life^2^.

Despite significant global progress have been made over the last two decades, severe food insecurity and undernourishment are still increasing in almost all regions of Africa^3^. According to the latest estimates of the State of Food Insecurity in the World 2021^4^, an estimated 720 to 811 million people faced hunger in 2020. The report also documents that more than one-third of the world’s undernourished are found in in Africa (282 million)^4^.

Hunger and food insecurity are major concerns in Ethiopia. The country began with food deficiency in the early 1970s. According to the Global Hunger Index^5^, Ethiopia ranks 90^th^ out of 116 countries and ranks among the world’s hungriest. The latest update of the 2022 *Global Report on Food Crises* says^6^, Ethiopia is projected to face one of the world's most severe food crises in 2022, resulting from the combined effects of escalating violence, prolonged drought and macroeconomic instability. The Productive Safety Net Program (PSNP), World Food Program (WFP) and government are struggling to alleviate the hunger crisis in Ethiopia, however, the combined effects of the COVID19 pandemic, locust invasions, floods, drought, market disruptions, and high food prices have left about 13.6 million people food insecure^7^.

The lack of progress towards the WHO global nutrition targets and the increase of severe food insecurity prevalence were major challenges for countries to create a world without hunger and malnutrition by 2030^8^. Identifying the possible factors that may affect severe food insecurity, is key to achieving the targets of Sustainable Development Goals (SDGs).

Findings from previous studies, highlight a number of factors that may affect the household food insecurity includes credit services^9–11^, age of the household^10,11^, gender of household head^12–14^, education of the household head^15–17^, family size^17,18^, household assets^13,19^ and dependency ratio^15,17,20^. Furthermore, severe food insecurity consequences may arise from uneven exposure burdens and differential susceptibility to exposure spatially^21–24^. These special factors have been shown to have a significant association with household food insecurity^23^. However, studies from Africa designed to adjust for these covariates are limited and there is no study that has assessed effect modification or interaction by these factors.

Mathematical models have been extensively used in research into household severe food insecurity dynamics because they play an important role in improving our understanding of major factors contributing to the household severe food insecurity dynamics. These models range from logistic regression^25–29^, linear regression model^30,31^, generalized linear mixed-effects models^32,33^, generalized estimating Equations^34,35^, to additive model^36^. However, these classical modelling approach, without considering spatial effect, affects statistical inference in many ways. Firstly, the classical model have deflated estimates of residual and variance^37^. This leads to higher type I error rates and loss of model precision^38^. Secondly, when predictor variables exhibit different degrees of autocorrelation and spatial patterns, the classical models produce overestimated statistical significance of the autocorrelated variables^39^. Lastly, failure to consider spatial autocorrelation may result in over-optimistic estimates of the model's predictive power and inappropriate model choice^40^. In addition, no previous study has directly analyzed the nonlinear effects of the covariates (i.e. age, family size, household assets and dependency ratio) on household severe food insecurity dynamics. Thus, the current study, using latest cohort big dataset, attempts to address these limitations and expands our understanding of the dynamics of household food insecurity in Ethiopia using the geo-additive model. The model assists to investigate factors that affect the severe household food insecurity by accounting for the spatial as well as the linear and the non-linear effects of demographic, economic and clinical factors.

## Methods

### Data description

**Figure 1:**
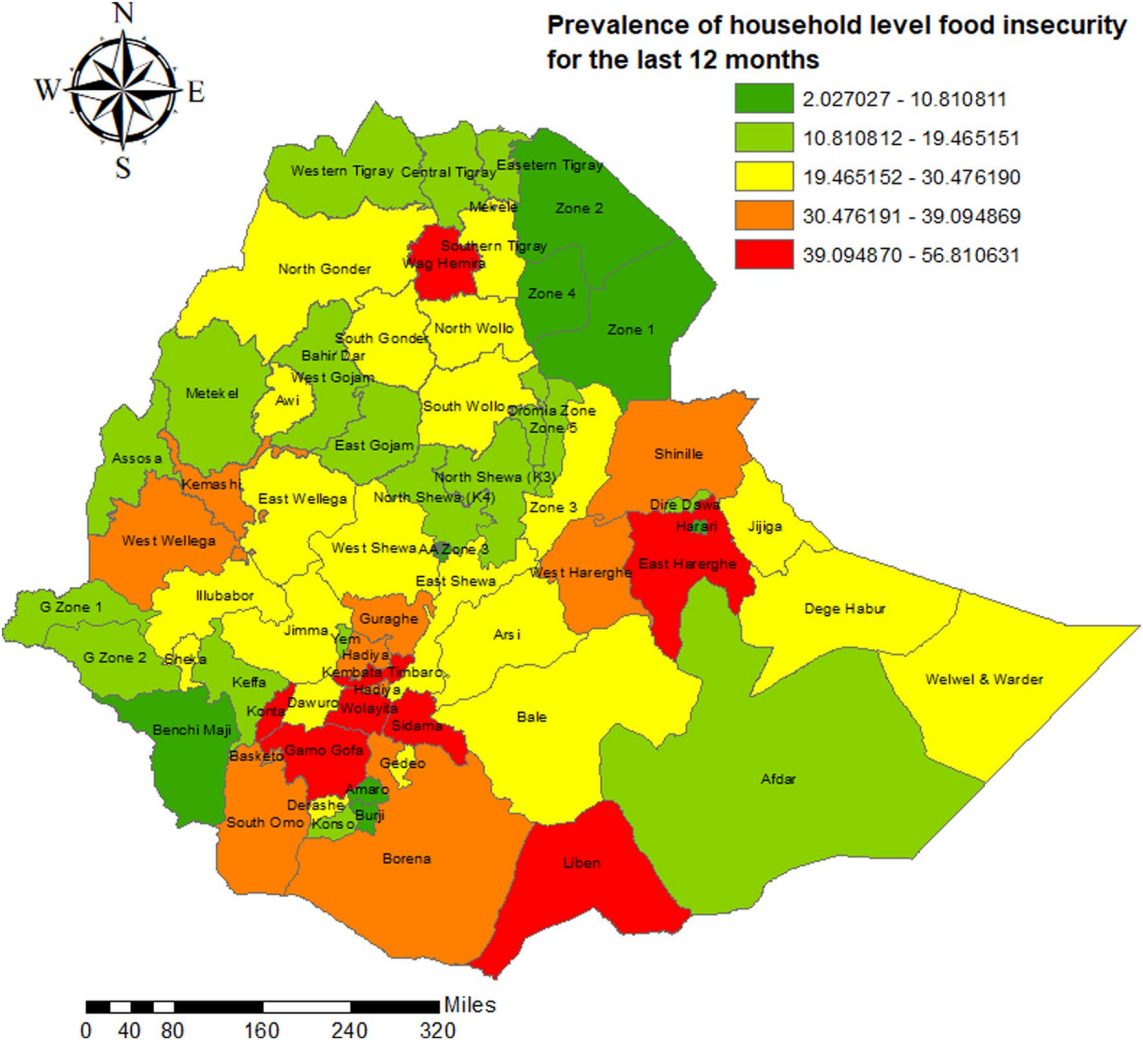
Observed household level food insecurity for the last 12 months across Ethiopia.

The data is from the Ethiopia Socioeconomic Survey (ESS) conducted by the ECSA (Ethiopian Central Statistics Agency) in collaboration with the World Bank. ESS is a long-term project to collect nationally representative panel survey of over 6500 households. The ESS collects information on economic activities along with other information on household agricultural activities, households like human capital, access to services and resources. The 1st wave was carried out in 2011/12, the 2nd wave in 2013/14, the 3rd wave in 2015/16 and the 4th wave was effected in 2018/19. The ESS sample is a two-stage probability sample. It employs a stratified, two-stage design where the regions of Ethiopia serve as the strata. The samples are regionally representative for the major regions of the country (Southern Nations & Nationalities Peoples (SNNP), Tigray, Amhara, Oromia, Afar and Somali) as well as Addis Ababa. For the purpose of this study, five thousand two hundred sixty-two (5262) households were included from 72 Ethiopian administrative zones over 8 years. The GPS coordinates for clusters were obtained from Ethiopian Demographic and Health Survey (EDHS) and those clusters were linked with the corresponding administrative zones for spatial analysis. The shapefiles for administrative zones were freely available on DIVA-GIS project website^41^. The map of the study sites was displayed in Fig. 1.

### Variables and measurements

For this study, the unit of analysis is households. Self-reported food insecurity status is used by recalling a situation of “household was worried that there would not be enough food in the past 7 days” and “food unavailability over the last 12 months”, prior to the interview. These outcome variables can reasonably address the four components of food security (i.e. Availability, Access, Utilization and Stability).

Household asset index is a composite indicator, calculated using PCA. It measures the ownership of a mobile phone, bed, gas cooker, bicycle, radio, motor bike, car, and sewing machine among others. Dependency ratio gives insight into the number of people of non-working age, compared with the number of those of working age. It is calculated as the ratio of nonworking age individuals to working age individuals. Working age individuals or producers are often defined as being 15–64 years of age, while those less than 15 years or older than 64 years are considered consumers. Moreover, household shocks measures whether the household had, in the past year, experienced any shocks, such as drought, death, theft, floods, illness, loss of jobs, violence, and local unrest.

### Generalized geo-additive mixed model

To assess the effects of factors on the household level food insecurity, we adopted a geo-additive model using Bayesian approach. This approach allows to simultaneously estimate the spatial effects of the geographical locations, unknown nonlinear effect of the covariates, as well as the vectors of the fixed effect parameters. This approach also provides monotonicity constraint estimates and allows to borrow extensions developed for Bayesian mean regression such as multilevel structures and regularization priors without the necessity to re-develop the complete inferential machinery^42,43^.

We first performed bivariate analysis to find out the determinants of household food insecurity. We only included variables significantly associated with severe food insecurity in the bivariate analysis in a multivariable analysis. For continuous variables, we checked for the shape of the relation using graphical smoothing techniques.

### Model specification

Let y_i_ be the status of food insecurity of household *i* (and is recoded as 1 if the household faced food insecurity, or 0 otherwise). The risk of a household food insecurity can be associated with head of household gender, age, average year of schooling in the household, family size, credit service, household assets and dependency ratio using a GLM model with the appropriate link function. GLM is flexible to allow for nonnormal outcome variable^44^. Thus, the probability of food insecurity for a household *i* in this study is defined as

1 $$\eta_{i} = log\left( {\frac{{\pi_{i} }}{{1 - \pi_{i} }}} \right) = \alpha_{0} + \alpha_{1} Sex + \alpha_{2} MS + \alpha_{2} CS + \alpha_{3} S + \alpha_{4} Age + \alpha_{5} AS + \alpha_{5} DR + \alpha_{6} Asset + \alpha_{7} FS$$

where ${\pi }_{i}$ is the probability of food insecurity for a household i, MS is household marital status, CS is credit services status (is recoded as 1 if the household uses credit service, or 0 otherwise), S is shock status (is recoded as 1 if the household have experienced any shocks, or 0 otherwise), AS is average years of schooling of all family members, DR is dependency ratio of the household and FS is family size of the household.

However, the standard GLM (Eq. 1) assume independent observations (or at least uncorrelated). But, this assumption is not always satisfied, sometimes observations exhibit temporal or/and spatial dependence. This variability has to be incorporated in the model. The predictors modified by taking into accounts the spatial auto-correlation and the non-linear effects of continuous covariates in the current study, can be given as follow

2 $$\eta_{i} = log\left( {\frac{{\pi_{i} }}{{1 - \pi_{i} }}} \right) = \alpha_{0} + \alpha_{1} Sex + \alpha_{2} MS + \alpha_{2} CS + \alpha_{3} S + f_{1} \left( {Age} \right) + f_{2} \left( {AS} \right) + f_{3} \left( {DR} \right) + f_{4} \left( {Asset} \right) + f_{5} \left( {FS} \right) + f_{6} \left( {uns\_spacial} \right) + f_{7} \left( {str\_spacial} \right)$$

where ${f}_{\mathrm{i}}(.)$ for *i* = 1,…,5 are the smooth functions expressing the unknown nonlinear effects of the covariates. ${f}_{6}(\mathrm{uns}\_\mathrm{spacial})$ is spatially unstructured random effects to capture the over dispersion at each location (i.e. regions in Ethiopia). ${f}_{7}(\mathrm{str}\_\mathrm{spacial})$ is structured correlated spatial effect to capture the unobserved spatial heterogeneity and to account the spatial autocorrelations. Equation (2) provides a class of models known as geo-additive model which is a more flexible semiparametric geo-additive regression model.

### Prior assumptions and inference

For implementation of our model (i.e. semiparametric geo-additive regression model), a Bayesian approaches which needs priors assumption have to be used. In Bayesian approach, all the smooth functions and the coefficients are assigned prior distributions. In the absence of any prior knowledge, diffuse prior (i.e. *p* (αi ∝ const) is the appropriate choice for fixed effect parameters^45,46^. For the smooth functions ${f}_{\mathrm{i}}(.)$ for i = 1,2,3,4,5, a 2nd order random walk prior was considered. Let x is covariate with equally spaced observations x i . Suppose that an ordered distinct values for a covariate are x(1) < ··· < x(t) < ··· < x(m) and define f(t) = f(x(t)). Therefore, the 2nd order random walk is given by f(t) = 2 f. (t−1)−f(t−2) + u(t) with are Gaussian errors (i.e. u(t)) and diffuse priors for initial values. For the spatially correlated effects, the nearest neighbor Gaussian Markov model was chosen^47^. For unstructured spatial effects, the parameters were assumed to be i.i.d. Gaussian with unknown variance^48^ (i.e. ${f}_{6}(uns\_spacial)\sim N(0, {\tau }^{2})).$ Therefore, highly dispersed inverse Gamma distributions are chosen.

For the analysis, we used the Markov Chine Monte Carlo algorithm to sample from the posterior conditional distributions. For each posterior distribution, we report the mean of the 1000 values as the parameter estimate, the SD as the measure of parameter variability, and the 2.5 and 97.5 quantiles, as the 95% credible interval. Model diagnosis was performed based on Markov Chine Monte Carlo postestimation diagnosis, besides the samples of the parameters, autocorrelation plots and implemented trace and, were also extracted using function sample. All of the analyses were implemented using R, version 4.0.5 and in order to map spatial effects, ArcGIS 10.6 was used.

## Results

The distribution of economic, demographic and clinical variables at baseline is presented in Table 1. The prevalence of food insecurity for female household in the sample was larger 29.8% compared to male household (26.3%). The prevalence of food insecurity was much higher among rural population, at 34.4%, compared with 15.2% among urban population. A similar difference was noted for food insecurity in the past 12 months, at 46.5% among household who have encountered shocks (natural or social) compared with 16.5% among household who haven’t encountered shocks. The prevalence of food insecurity for Somalia region (39.0%), Benshagul-Gumuz (35.2) and SNNP (35.2%) has accounted for relatively higher portion compared to other regions in Ethiopia. Chi-square test indicated that there is a significant association among geographic variables; residence, region and food insecurity in the past 12 months was found (*p*-value < 0.0001). Furthermore, chi-square test also indicated that household food insecurity was significantly associated with sex, marital status, shocks and availability of credit services (*p*-value < 0.05).

**Table 1:** Association between household level food insecurity and geographic, demographic and socio-economic variables.

Variables	Food insecurity for the last 12 months		Total	Chi-square/Independent t-test *P*-value
	No	Yes		
**Gender, n (%)**				
Male	2690 (73.7%)	958 (26.3%)	3662 (69.6%)	0.009**
Female	1120 (70.2%)	475 (29.8%)	1599 (30.4%)	
**Marital status, n (%)**				
Never Married	363 (87.5%)	52 (12.5%)	416 (8.0%)	0.000***
Married	2601 (73.2%)	953 (26.8%)	3568 (68.2%)	
Divorced/ Separeted	339 (67.4%)	164 (32.6%)	504 (9.6%)	
Widowed	488 (65.9%)	253 (34.1%)	743 (14.2%)	
**Region, n (%)**				
Tigray	490 (79.9%)	123 (20.1%)	613 (11.6%)	0.000***
Afar	106 (78.5%)	29 (21.5%)	136 (2.6%)	
Amhara	727 (70.6%)	303 (29.4%)	1034 (19.7%)	
Oromia	779 (74.1%)	272 (25.9%)	1056 (20.1%)	
Somalia	177 (61.0%)	113 (39.0%)	290 (5.5%)	
Benshagul-Gumuz	81 (64.8%)	44 (35.2%)	125 (2.4%)	
SNNP	772 (64.8%)	419 (35.2%)	1194 (22.7%)	
Gambelia	110 (86.6%)	17 (13.4%)	130 (2.5%)	
Harari	143 (87.2%)	21 (12.8%)	165 (3.1%)	
Addis Ababa	275 (92.6%)	22 (7.4%)	297 (5.6%)	
Diredwa	150 (67.9%)	71 (32.1%)	222 (4.2%)	
**Residence, n (%)**				
Rural	2168 (65.6%)	1139 (34.4%)	3323 (63.2%)	0.000***
Urban	1642 (84.8%)	295 (15.2%)	1939 (36.8%)	
Shocks, n(%)				
Yes	1014 (53.5%)	882 (46.5%)	1906 (36.2%)	0.000***
No	2796 (83.5%)	552 (16.5%)	3356 (63.8%)	
**HH with access to credit, n (%)**				
Yes	2975 (75.9%)	484 (36.9%)	1321 (25.2%)	0.000***
No	2690 (73.7%)	944 (24.1%)	3929 (74.8%)	
Household size, mean(± SD)	4.32 (2.33)	4.59 (2.43)	4.36 (2.35)	0.000***
Average years of schooling in the HH, mean(± SD)	7.27 (4.47)	4.89 (3.20)	6.99 (4.41)	0.000***
Dependency ratio, mean(± SD)	0.84 (0.87)	1.14 (1.01)	0.88 (0.90)	0.000***
Asset, mean(± SD)	3.11 (1.42)	2.31 (1.18)	3.00 (1.41)	0.000***
Age, mean(± SD)	41.85 (15.03)	44.44 (15.51)	42.19 (15.12)	0.000***

**Figure 2:**
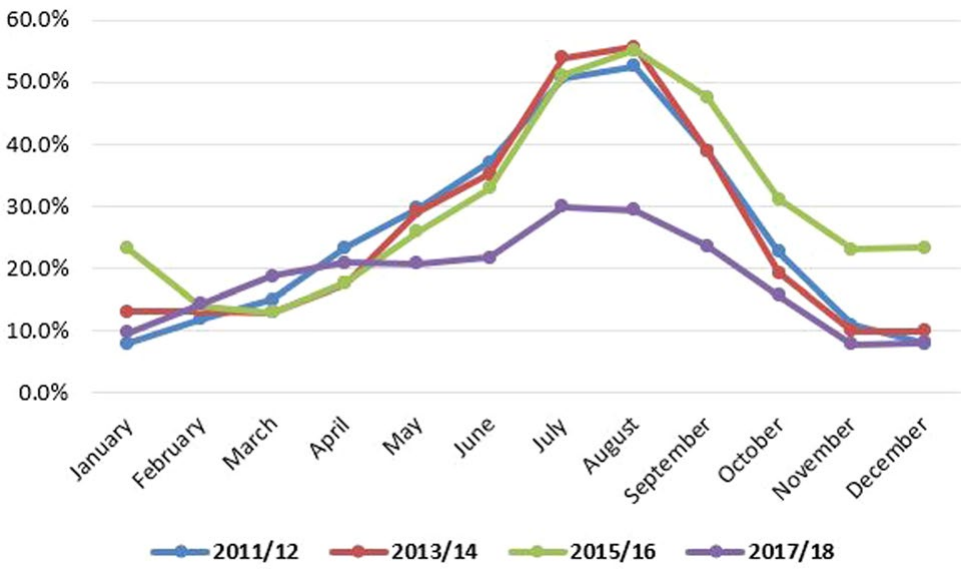
Dynamics of household level food insecurity over the months.

Figure 1 shows the prevalence of household level food insecurity across all zones in Ethiopia. It clearly demonstrated that the Wag Hemira, East Harerghe, Liben, Gamo Gofa, Sidama and Wolayta zones registered higher prevalence of household level food insecurity than the other zones in Ethiopia. Though the prevalence of household level food insecurity is more pronounced in rural populations, the months of August, July, June, and September are identified as slack periods for many households (Fig. 2).

The overall mean household size was 4.36 ± 2.35 with a higher mean household size for those who are food insecure (4.59 ± 2.43) compared to those who are not food insecure (4.32 ± 2.33). Similarly, among the household who are food insecure, the average years of schooling in the household, dependency ratio, asset and age were 4.89 ± 3.20, 1.14 ± 1.01, 2.31 ± 1.18 and 44.44 ± 15.51 years, respectively while the average years of schooling in the household, dependency ratio, asset and age of household who are not food insecure were 7.27 ± 4.47, 0.84 ± 0.87, 3.11 ± 1.42 and 41.85 ± 15.03 years, respectively. The mean difference was also statistically significant at 5% significance level.

### Predictors of household level food insecurity

#### Assessment of the fitted model

All the goodness of fit assessment results showed that the final model fitted the data adequately. Between the two models above (Eqs. 1 and 2), the generalized geo-additive mixed model given in Eq. (2) resulted in the lowest DIC, which confirms the appropriateness of the model. In the selected model, predictor variables that was significant at a 10% level in the single covariate analysis were included in the multivariable model^49^. Furthermore, the range of the estimated structured spatial effect was much higher than the range of random spatial effect, indicating that model fit improvement by including structured spatial effects, is relatively greater.

#### Spatial effects

**Figure 3:**
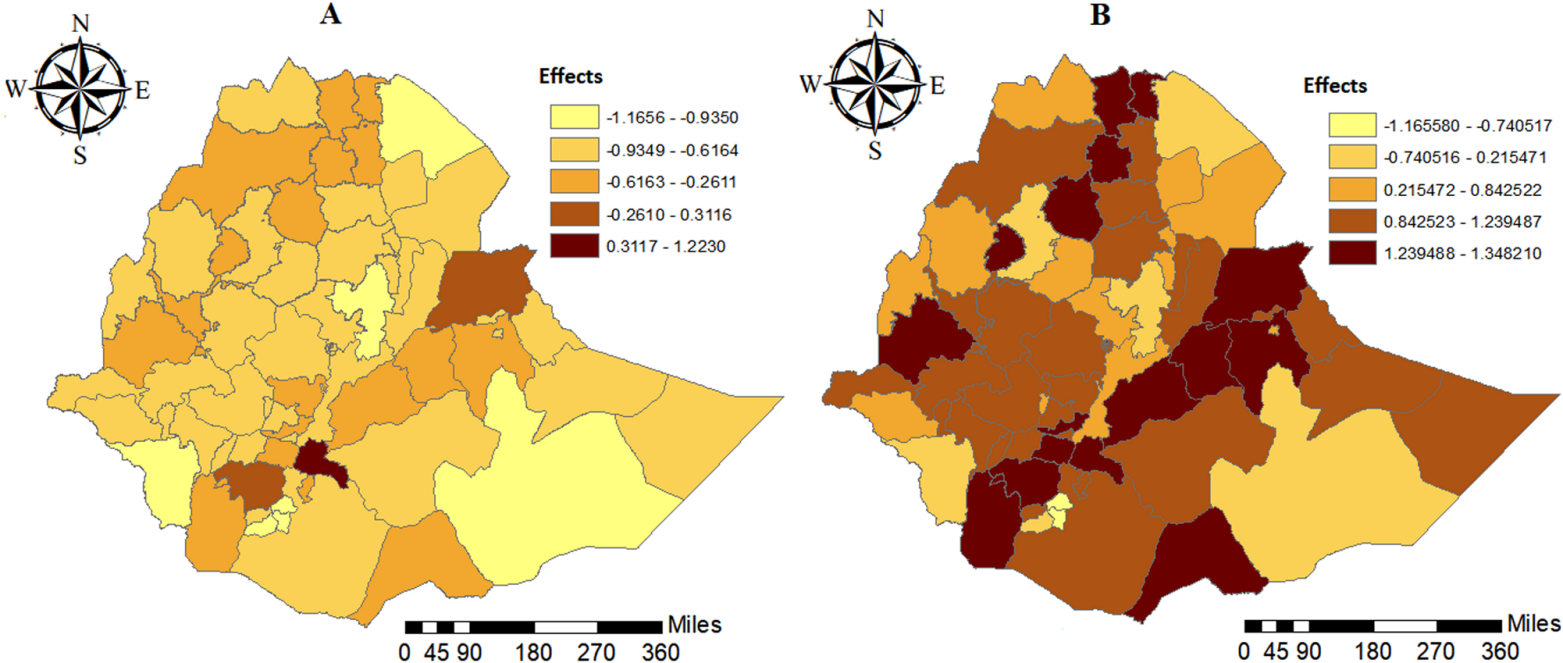
Estimated mean effect: (**A**) unstructured spatial effect; (**B**) the structured spatial effect.

The spatial effects presented in Fig. 3 is based on generalized geo-additive mixed model. Our model results showed both negative and positive spatial effects on food insecurity among the households in Ethiopia. The variance estimates for the cluster-level spatial effects (which account for the spatial autocorrelation) and the zone-level random effect were nonzero and significant (Table 2).

**Table 2:** Estimated parameters (with 95% CI) of geo-additive model for non-linear effects on the household level food insecurity.

Variables	Coefficient	95% CI	
		Lower	Upper
Age	0.022	0.001	0.128
Average year of schooling in HH	0.018	0.001	0.140
Asset	0.097	0.011	0.554
Dependency ratio	0.038	0.002	0.310
Household size	0.059	0.003	0.363

The colors on the maps (Fig. 3B) ranged from yellow to dark red such that the dark red and brown colors denoting a positive effect which are associated with an increased risk of severe food insecurity (i.e.), while the yellow and orange color show the negative effects on household level food insecurity. Moreover, the map showed that the presence of variation in household level food insecurity in Ethiopian clusters. Mainly, household living in the Sidama, Gamo Gofa, Shinille, Basketo, Wolyita, Wag Hemira, Liben, Awi, Eastern Tigray and West Harerghe zones, showed higher food insecurity than the other zones in Ethiopia (Fig. 3B). Geographically, these areas are generally characterized cash crop producers, densely populated, high climate vulnerability and undulating hills limited agricultural production potential that barely produced enough food to meet the food demands of households.

#### Fixed effects

**Figure 4:**
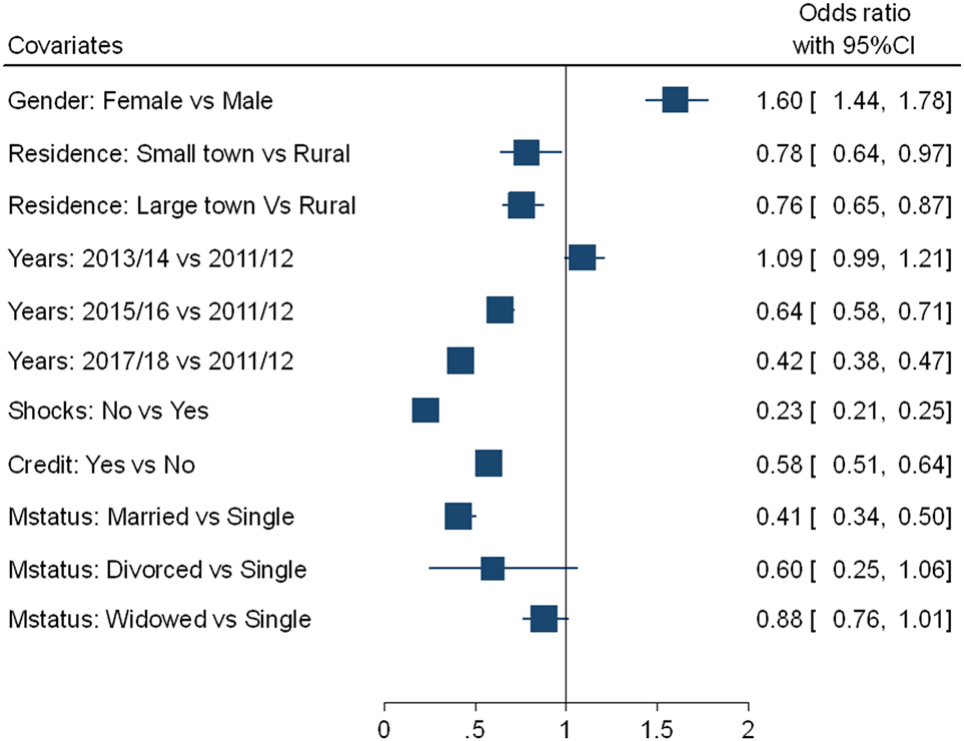
Estimated parameters (with 95% CI) of geo-additive model for linear effects on the household level food insecurity.

**Figure 5:**
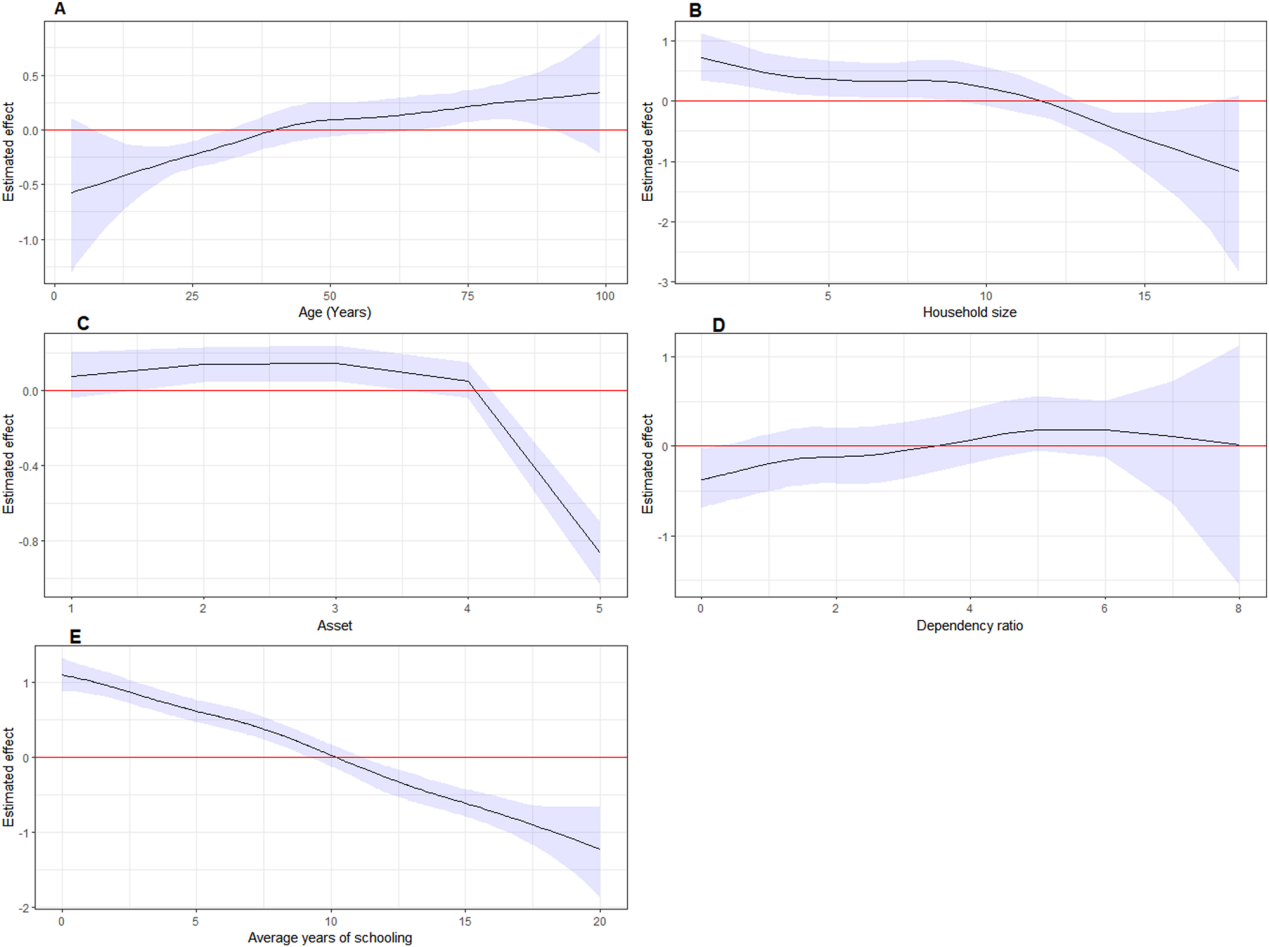
Non-linear effects of continuous predictors (with 95% CI) of geo-additive model: (**A**) Age of the household; (**B**) Household size; (**C**) Household assets index; (**D**) dependency ratio; (**E**) Average years of schooling of all family members.

Analysis results for modeling the dynamics of household level food insecurity using generalized geo-additive mixed model are presented in Figs. 4 and 5. The residuals plots of the geo-additive model also showed no discernible patterns. Based on the results, we observed that the female-headed households (aOR = 1.60, 95%CI: 1.44–1.78) tend to have a higher probability of being food-insecured, compared to male-headed households. Households living in small town and large town have 22% (aOR = 0.78, 95%CI: 0.64–0.97) and 24% (aOR = 0.76, 95%CI: 0.65–0.87) lower probability of being food-insecured, respectively than households living in rural areas. Furthermore, households who reported married are significantly associated with a lower probability of being food-insecured (aOR = 0.41, 95%CI: 0.34–0.50), compared to single-headed households.

Households who did not face any shocks (natural or social) in the last 12 months periods, have 77% lower probability of being food-insecure (aOR = 0.23, 95%CI: 0.21–0.25) than those who have encountered any such shocks. Households with access to credit service have 42% (aOR = 0.58, 95%CI: 0.51–0.64) lower probability of being food-insecure compared to those without the services. Considering time effects of food insecurity, a decreased likelihood of being food-insecure in 2015/16 (aOR = 0.64, 95%CI: 0.58–0.71) and 2017/18 (aOR = 0.42, 95%CI: 0.38–0.47), compared to 2012/13.

#### Non-linear effects

The non-linear effect of continuous variables on household level food insecurity is given in Fig. 5 and Table 2. Based on the findings, we noted that age of the household had a significant non-linear effect on food insecurity (Fig. 5A). The lowest food insecurity is observed at age below 40 years. Thereafter, an increasing risk of food insecurity for age greater than 40 years. On the other hand, household asset index in Fig. 5C, illustrates an association with the risk of food insecurity. The Figure further suggested that a minimal effect with household asset index less than 4 and subsequently a decreasing risk of food insecurity for household asset index greater than 4. Figure 5B shows a decrease with the risk of food insecurity as the number of family size increases in the household. Figure 5E demonstrates a steady drop in the risk of food insecurity with increasing average year of schooling of all family members. Furthermore, the lowest food insecurity is observed for dependency ratio less than 3. Thereafter, a minimal positive effect with dependency ratio greater than 3 (Fig. 5D).

## Discussion

The current study investigated the spatial heterogeneity of household level food insecurity, and its association with head of household gender, age, average year of schooling in the household, family size, credit service, household assets and dependency ratio. Several researchers have studied the determinants of household level food insecurity in Africa^26,33,50–54^ and their findings supports the factors obtained in this study. However, the extra regional and possible intra-regional spatially modified effect of predictors of household level food insecurity has not been previously described. For this reason, the geoadditive model that includes the structured and unstructured special effects have been used in the current study. A model that allows unknown non-linear relationship b/n the response variable and continuous variables is very important. In addition to the non-linear effect, predictors modified by taking into accounts the spatial auto-correlation, leads to more accurate estimates of the determinants of household level food insecurity. Consequently, the findings of the current study are expected to lead to better estimates of the factors. To our knowledge, no other African study has investigated these spatial effects on of household level food insecurity.

Some of the results of the current study supported the previous literature, whilst some of the results provided new insights. The findings of the current study revealed that the highest prevalence of household food insecurity was observed from the lowland, hilly and mountainous highlands, which is household living in the Eastern Tigray, Sidama, Wag Hemira, Liben, Shinille, Gamo Gofa, Basketo, Wolyita, Awi, and West Harerghe zones. A plausible reason for the high food insecurity Eastern Tigray, Wag Hemira and Awi zones lower crop production potential due to its lower temperature, unfavorable agroecologic conditions, limited access to infrastructure and low natural resource endowments^55^. The high rates of food insecurity in Shinille, Liben and Western Hararghe which are in Somali region may be attributed to because 2015 El-Nino drought that affected food securities in the Somali because of significant rainfall decrement and many livestock deaths in pastoralists^56^. The three study zones, Sidama, Wolayta and Gamo Gofa were in the SNNP region where Sidama well known for its coffee production, grown as a cash crop. Wolayta and its neighbor Gamo Gofa are densely populated zone where land shortage is a major problem^57^. Therefore, giving special attention for these areas while designing interventions are required.

Female-headed households were also more likely prone to food insecurity compared to male-headed households. This was supported by previous studies Sisha^33^, Agidew and Singh^54^, Jung, et al.^14^, and Negesse, et al.^12^. This could be possibly explained by the fact that female-headed households specially from the rural areas cannot adopt new agricultural technologies^58^ and also faced multiple challenges such as lack of agriculture extension services and limited access to land ownership^59^. Old household heads were significantly associated with an increased likelihood of experiencing household level food insecurity. This was supported by previous findings^54,60,61^. They found that an increase age of household head decreases the probability of being food-secure.

Schooling of members of the household negatively affects household food insecurity. This is consistent with other research papers that demonstrated food insecurity to be significantly correlated with education^33,62,63^. This could be possibly explained by the facts that educational attainment by the household members largely contributed on working competency, diversify income, efficiency, and adopting technologies with long term target to ensure better living condition. A study conducted in Ethiopia^64,65^, Kenya^63^ and Bangladesh^66^ also supported the above conclusion. Furthermore, education is associated with better job opportunities and provides households with the knowledge of how to meet nutritional needs and health of their families.

Households with access to credit services in the current study were associated with reduced probability of food insecurity. Evidence has shown that access to credit had a positive influence on a household’s food security^54,67–69^. This could be due to the facts that, particularly rural area, access to credit enables households to make timely purchase of inputs (agricultural), such as improved seeds, fertilizer, herbicides, and pesticides, which in turn enhances farm productivity and increases consumption expenditure and future food availability. Therefore, access to credit services supports households to put themselves at a better status of food security. In addition, It is an essential component of development in any economy. On the other hand, households with higher wealth are associated with reduced probability of food insecurity, a result consistent with earlier studies^70,71^.

Our analysis also showed that having a high dependency ratio was significantly associated with an increased likelihood of food insecurity. This is consistent with other findings which argue that households having old age groups and many children may lack sufficient manpower, which eventually results in overdependence on limited family resources hence food insecurity^33,54,61,72^. Thus, promoting family planning helps women limit the number of their children and hence reduces the probability of household level food insecurity.

Shocks coming from various causes are expected to affect vulnerable population. Our analysis also showed that the presence of shock was an independent predictor of food insecurity among households across all rural, semi-urban and urban residences. A study carried out by Sisha^33^, Owoo^73^ and Gupta, et al.^74^ also came up with similar findings. They found that presence of economic shocks such as droughts, floods, or changes in incomes and price, among others, households have worse food security outcome.

## Conclusions

Overall, from a methodological perspective, our ability to include extra and intra-regional spatially modified effects on household level food insecurity, adjust for several household-level factors known to be associated with food insecurity, and incorporate these into semi-parametric geo-additive model, strengthened our findings over previous studies. Our findings lay the foundation for future work to identify specific directions for intervention. Identifying regions that are likely to have chronic food insecurity helps to take measures in those regions that require special attention. This study also proved that food insecurity continued to be a problem for the Ethiopian population. The findings of this study contribute to a better understanding of the spatial distribution of chronic food insecurity and together with possible factors that affect chronic food insecurity among adolescents.

## Data Availability

The dataset used and analyzed during the current study is available from the corresponding author on reasonable request.

## References

[1] (FAO), F. A. O. o. t. U. N. The state of food insecurity in the world 2014*: Strengthening the enabling enviroment for food security and nutrition*. (Food & Agriculture Org, 2015).

[2] Anderson, S. & Farmer, E. USAID Office of food for peace food security country framework for Ethiopia FY 2016–FY 2020. *Washington, DC: Food Economy Group*, 13 (2015).

[3] UNICEF & WHO. The state of food security and nutrition in the world 2017: Building resilience for peace and food security. (2017).

[4] Organization, W. H. The state of food security and nutrition in the world 2021*: Transforming food systems for food security, improved nutrition and affordable healthy diets for all*. Vol. 2021 (Food & Agriculture Org., 2021).

[5] Index, G. H. 2021-Global hunger index-Ethiopia policy brief. (2022).

[6] Crises, G. N. A. F. Global Report on Food Crises: Joint analysis for better decisions. *Rome* (2022).

[7] WFP. Millions could fall deeper into hunger as WFP faces unprecedented funding Gap in Ethiopia. (2021).

[8] ECA, F. Regional overview of food security and nutrition: Addressing the threat from climate variability and extremes for food security and nutrition. *Food Agric. Organ. United Nations*, 116 (2018).

[9] Habtewold TM (2018). in Economic growth and development in Ethiopia 39–65.

[10] Mohammed A, Wassie SB, Teferi ET (2021). Determinants of smallholders’ food security status in kalu district, Northern Ethiopia. Challenges.

[11] Sani S, Kemaw B (2019). Analysis of households food insecurity and its coping mechanisms in Western Ethiopia. Agric. Food Econ..

[12] Negesse A (2020). The impact of being of the female gender for household head on the prevalence of food insecurity in Ethiopia: A systematic-review and meta-analysis. Public Health Rev..

[13] Zhou D (2019). Factors affecting household food security in rural northern hinterland of Pakistan. J. Saudi Soc. Agric. Sci..

[14] Jung NM, de Bairros FS, Pattussi MP, Pauli S, Neutzling MB (2017). Gender differences in the prevalence of household food insecurity: A systematic review and meta-analysis. Public Health Nutr..

[15] Mengistu, S. W. & Kassie, A. W. Household level determinants of food insecurity in rural ethiopia. *J. Food Quality* **2022** (2022).

[16] Dula T, Berhanu W (2019). Determinants of rural household food security and coping up mechanisms in the case of woliso woreda western Ethiopia. World J. Agric. Soil Sci..

[17] Derso A, Bizuneh H, Keleb A, Ademas A, Adane M (2021). Food insecurity status and determinants among urban productive safety net program beneficiary households in addis ababa Ethiopia. PLoS ONE.

[18] Liao JM, Navathe AS (2018). Nudging physicians to reduce quetiapine prescribing using medicare letters: Following the letters of the law?. JAMA Psychiat..

[19] Masa R, Chowa G, Nyirenda V (2017). Prevalence and predictors of food insecurity among people living with HIV enrolled in antiretroviral therapy and livelihood programs in two rural Zambian hospitals. Ecol. Food Nutr..

[20] Samim SA (2021). Food insecurity and related factors among farming families in Takhar region Afghanistan. Sustainability.

[21] Charreire H (2010). Measuring the food environment using geographical information systems: A methodological review. Public Health Nutr..

[22] Bivoltsis A (2018). Food environments and dietary intakes among adults: does the type of spatial exposure measurement matter? A systematic review. Int. J. Health Geogr..

[23] Schuette CK, Laninga T (2016). The spatial distribution and quantification of food insecurity in the north central health district of Idaho. J. Hunger Environ. Nutr..

[24] Tomita A (2020). Spatial clustering of food insecurity and its association with depression: a geospatial analysis of nationally representative South African data, 2008–2015. Sci. Rep..

[25] Shah SK (2020). Multinomial logistic regression model to identify factors associated with food insecurity in rural Households in Nepal. Nepalese J. Stat..

[26] Getacher L (2020). Food insecurity and its predictors among lactating mothers in North Shoa Zone, Central Ethiopia: A community based cross-sectional study. BMJ Open.

[27] Tarasuk V, St-Germain A-AF, Mitchell A (2019). Geographic and socio-demographic predictors of household food insecurity in Canada, 2011–12. BMC Public Health.

[28] Gebrie YF (2021). Bayesian regression model with application to a study of food insecurity in household level: a cross sectional study. BMC Public Health.

[29] Gholami A, Sani TR, Askari M, Jahromi ZM, Dehghan A (2013). Food insecurity status and associated factors among rural households in North-East of Iran. Int. J. Prev. Med..

[30] Ogunniyi AI (2021). Socio-economic drivers of food security among rural households in Nigeria: Evidence from smallholder maize farmers. Soc. Ind. Res..

[31] Phami P (2020). Exploring the determinants of food security in the areas of the Nam Theun2 hydropower project in Khammuan Laos. Sustainability.

[32] Opsomer JD, Jensen HH, Pan S (2003). An evaluation of the US Department of Agriculture food security measure with generalized linear mixed models. J. Nutr..

[33] Sisha TA (2020). Household level food insecurity assessment: Evidence from panel data Ethiopia. Sci. African.

[34] Maes KC, Hadley C, Tesfaye F, Shifferaw S, Tesfaye YA (2009). Food insecurity among volunteer AIDS caregivers in Addis Ababa, Ethiopia was highly prevalent but buffered from the 2008 food crisis. J. Nutr..

[35] Maes KC, Hadley C, Tesfaye F, Shifferaw S (2010). Food insecurity and mental health: Surprising trends among community health volunteers in Addis Ababa, Ethiopia during the 2008 food crisis. Soc. Sci. Med..

[36] Magaña-Lemus D, Ishdorj A, Rosson CP, Lara-Álvarez J (2016). Determinants of household food insecurity in Mexico. Agri. Food Econ..

[37] Legendre P, Fortin MJ (1989). Spatial pattern and ecological analysis. Vegetatio.

[38] Beale CM, Lennon JJ, Yearsley JM, Brewer MJ, Elston DA (2010). Regression analysis of spatial data. Ecol. Lett..

[39] Lennon JJ (2000). Red-shifts and red herrings in geographical ecology. Ecography.

[40] Telford R, Birks H (2005). The secret assumption of transfer functions: problems with spatial autocorrelation in evaluating model performance. Quatern. Sci. Rev..

[41] Hijmans, R. J. DIVA-GIS version 5.2 manual diva http://www-gis. org (2005).

[42] Fahrmeir, L., Kneib, T. & Lang, S. Penalized structured additive regression for space-time data: A bayesian perspective. *Stat. Sinica*, 731–761 (2004).

[43] Kammann E, Wand MP (2003). Geoadditive models. J. Roy. Stat. Soc. Ser. C (Appl. Stat.).

[44] McCullagh, P. & Nelder, J. Generalized linear models., 2nd edn.(Chapman and Hall: London). *Standard book on generalized linear models* (1989).

[45] Brezger A, Kneib T, Lang S (2003). BayesX: Analysing Bayesian structured additive regression models.

[46] Fahrmeir L, Lang S (2001). Bayesian inference for generalized additive mixed models based on Markov random field priors. J. Roy. Stat. Soc. Ser. C (Appl. Stat.).

[47] Rue H, Held L (2005). Gaussian Markov random fields: theory and applications.

[48] Kneib T, Fahrmeir L (2007). A mixed model approach for geoadditive hazard regression. Scand. J. Stat..

[49] Wand H, Whitaker C, Ramjee G (2011). Geoadditive models to assess spatial variation of HIV infections among women in local communities of Durban, South Africa. Int. J. Health Geogr..

[50] Mota AA, Lachore ST, Handiso YH (2019). Assessment of food insecurity and its determinants in the rural households in Damot Gale Woreda, Wolaita zone, southern Ethiopia. Agri Food Secur..

[51] Abegaz KH (2017). Determinants of food security: Evidence from ethiopian rural household survey (ERHS) using pooled cross-sectional study. Agri. Food Secur..

[52] Fraval S (2019). Food access deficiencies in sub-Saharan Africa: Prevalence and implications for agricultural interventions. Front. Sustain. Food Syst..

[53] Drammeh W, Hamid NA, Rohana A (2019). Determinants of household food insecurity and its association with child malnutrition in Sub-Saharan Africa: A review of the literature. Curr. Res. Nutr. Food Sci. J..

[54] Agidew A-MA, Singh K (2018). Determinants of food insecurity in the rural farm households in South Wollo Zone of Ethiopia: The case of the Teleyayen sub-watershed. Agri. Food Econ..

[55] Alemu ZA, Ahmed AA, Yalew AW, Simanie B (2017). Spatial variations of household food insecurity in East Gojjam Zone, Amhara Region, Ethiopia: Implications for agroecosystem-based interventions. Agri. Food Secur..

[56] Derseh NM, Gelaye KA, Muluneh AG (2021). Spatial patterns and determinants of undernutrition among late-adolescent girls in Ethiopia by using Ethiopian demographic and health surveys, 2000, 2005, 2011 and 2016: A spatial and multilevel analysis. BMC Public Health.

[57] Gurmu, F., Hussein, S. & Laing, M. Diagnostic assessment of sweetpotato production in Ethiopia: Constraints, post-harvest handling and farmers' preferences. *Res. Crops* **16** (2015).

[58] Menale K, Ndiritu SW, Stage J (2014). What determines gender inequality in household food security in Kenya? application of exogenous switching treatment regression. World Dev..

[59] Quisumbing AR, Pandolfelli L (2010). Promising approaches to address the needs of poor female farmers: Resources, constraints, and interventions. World Dev..

[60] Bashir, M., Schilizzi, S. & Pandit, R. The determinants of rural household food security: The case of landless households of the Punjab. *Pa istan, Wor ing Paper* **1208** (2012).

[61] Ahmed FF, Abah PO (2014). Determinants of food security among low-income households in Maiduguri metropolis of Borno State, Nigeria. Asian J. Soc. Sci. Hum..

[62] Joshi N, Raghuvanshi RS (2020). Determinants of household food insecurity in rural areas of the Hilly Region of Kumaun, Uttarakhand, India: A pilot study. Ecol. Food Nutr..

[63] Mutisya M, Ngware MW, Kabiru CW, Kandala N-B (2016). The effect of education on household food security in two informal urban settlements in Kenya: A longitudinal analysis. Food Secur..

[64] Endale W, Mengesha ZB, Atinafu A, Adane AA (2014). Food insecurity in Farta district, Northwest Ethiopia: A community based cross–sectional study. BMC. Res. Notes.

[65] Regassa N, Stoecker BJ (2012). Household food insecurity and hunger among households in Sidama district, southern Ethiopia. Public Health Nutr..

[66] Faridi, R. & Wadood, S. N. An econometric assessment of household food security in Bangladesh.* Bangladesh Dev. Stud.*, 97–111 (2010).

[67] Frimpong S, Asuming-Brempong S (2013). Comparative study of determinants of food security in rural and urban households of Ashanti region Ghana. Int. J. Econ. Manag. Sci..

[68] Holden S, Shiferaw B (2004). Land degradation, drought and food security in a less-favoured area in the Ethiopian highlands: A bio-economic model with market imperfections. Agric. Econ..

[69] Nkomoki W, Bavorová M, Banout J (2019). Factors associated with household food security in Zambia. Sustainability.

[70] Mwangi V (2020). Linking household food security and food value chains in North West Mt Kenya. Sustainability.

[71] Hunnes DE (2015). The effects of weather, household assets, and safety-net programs on household food security in Ethiopia using rural household panel data. Reg. Environ. Change.

[72] Aidoo, R., Mensah, J. O. & Tuffour, T. Determinants of household food security in the Sekyere-Afram plains district of Ghana. *Euro. Sci. J.* **9** (2013).

[73] Owoo NS (2021). Demographic considerations and food security in Nigeria. J. Soc. Econ. Develop..

[74] Gupta P, Singh K, Seth V, Agarwal S, Mathur P (2015). Coping strategies adopted by households to prevent food insecurity in urban slums of Delhi India. J. Food Secur..

